# Conservative treatment for coronal shear fracture of the distal humerus: a case report

**DOI:** 10.1016/j.xrrt.2022.06.008

**Published:** 2022-07-30

**Authors:** Yuzuru Nakamura, Kenji Tsubo, Norihiro Sasaki, Nana Ichikawa, Harehiko Tsukada, Yoshihito Yamasaki, Yasuyuki Ishibashi

**Affiliations:** a Department of Orthopaedic Surgery, Hirosaki University Graduate School of Medicine, Hirosaki, Japan; b Department of Orthopaedic Surgery, Fuyoukai Murakami Hospital, Aomori, Japan; c Department of Orthopaedic Surgery, Aomori City Hospital, Aomori, Japan

**Keywords:** Coronal shear fractures, Distal humerus, Conservative treatment, Orthopedic surgery

Coronal shear fractures of the distal humerus are rare. They are usually the result of a low-energy fall and direct compression of the distal humerus by the radial head in a hyper-extended or semi-flexed elbow or from the spontaneous reduction of posterolateral subluxation or dislocation.^12^ Dubberley et al proposed a treatment- and outcome-oriented classification of capitellum and trochlea fractures: type 1 involves the capitellum with or without the lateral trochlear ridge, type 2 involves the capitellum and trochlea as one piece, and type 3 injuries consist of fractures of both the capitellum and the trochlea as separate fragments. These fractures were further characterized with respect to the absence (A) or presence (B) of posterior condylar comminution.^1^ Dubberley’s classification influences the choice of fixation method and outcome.^10^ Most of these fractures are displaced, and nonsurgical management results in multiple complications, including chronic pain, mechanical symptoms, and instability. Nevertheless, good-to-excellent outcomes can be achieved in most patients with open reduction and internal fixation.^3^

There are few reports regarding Dubberley’s type 2A coronal shear fractures of the distal humerus. To our knowledge, there are no reports of conservative treatment with closed reduction and casting. Therefore, we report a case of Dubberley’s type 2A fracture treated conservatively in a 17-year-old male patient. Informed consent was obtained from the patient and his family.

## Case report

**Figure 1:**
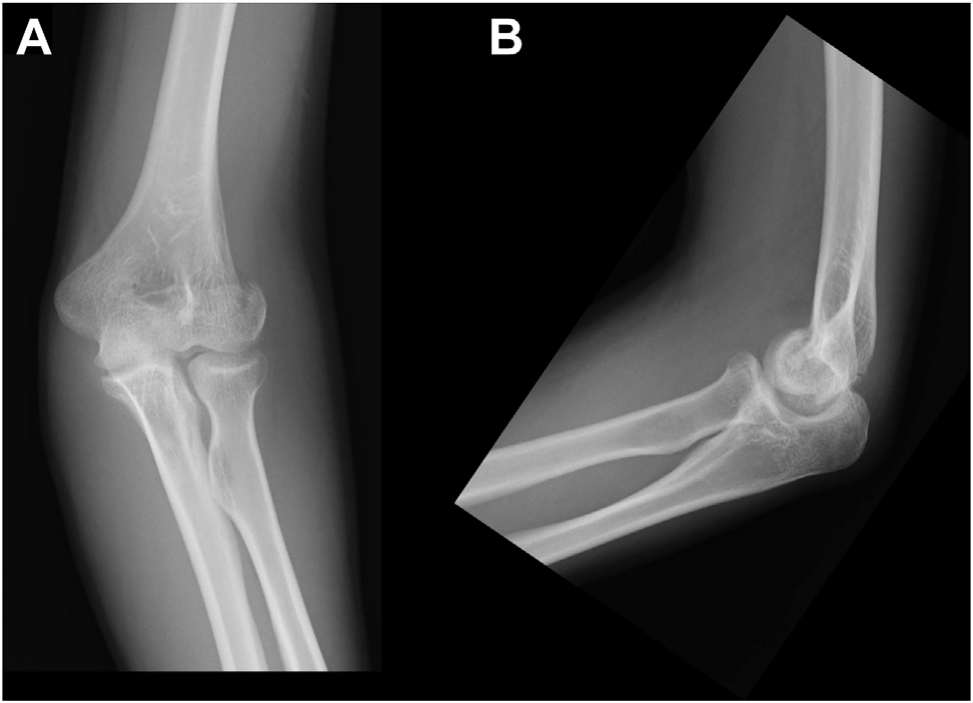
Plain radiograph of the elbow joint at the first visit to our hospital. (**A**) Anterior-posterior view and (**B**) lateral view. Coronal shear fracture of the distal humerus is observed. The bone fragment was migrated to the proximal side.

**Figure 2:**
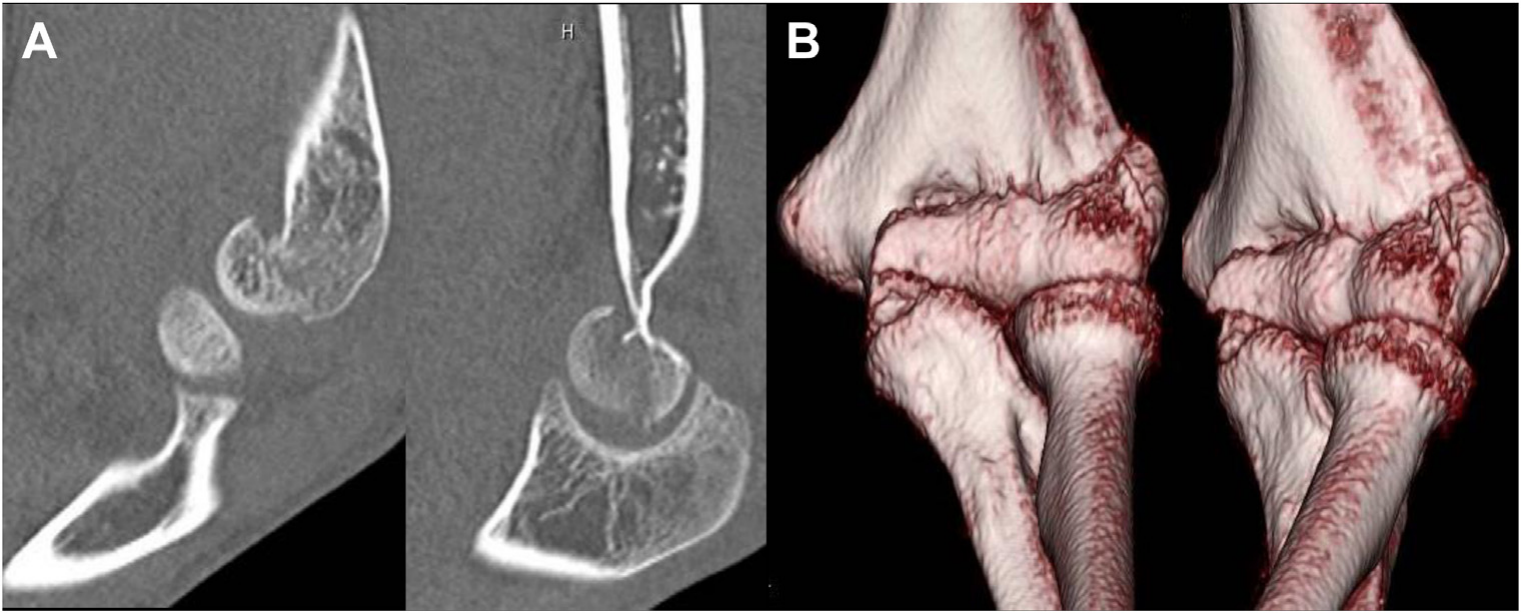
Computed tomography of the elbow joint at the first visit to our hospital. (**A**) Sagittal image and (**B**) 3-dimensional image. The distance of dislocation was 4 mm to the proximal side. The posterior humeral cortex was intact.

**Figure 3:**
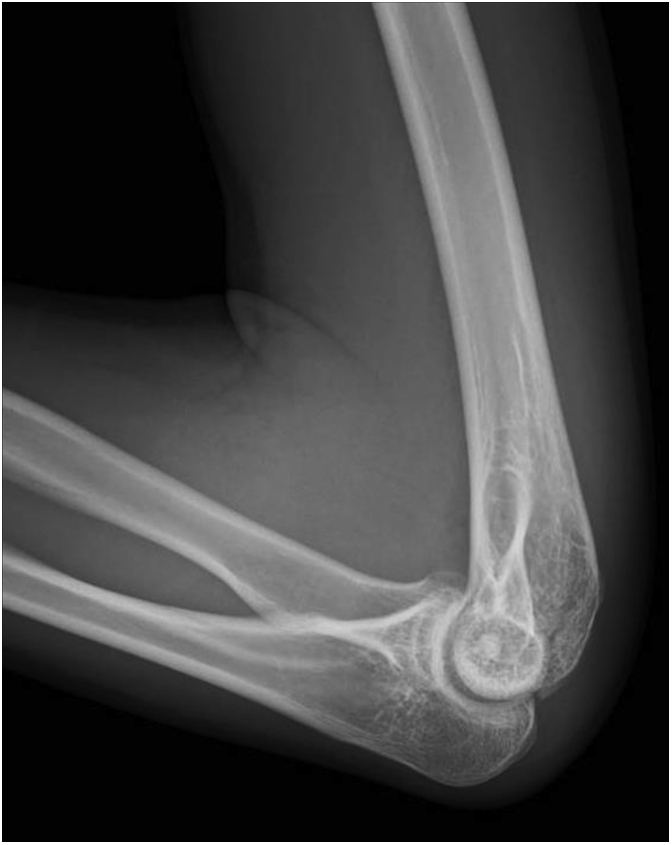
Plain radiograph of the elbow joint after reduction. Lateral view. An anatomical reduction was obtained.

**Figure 4:**
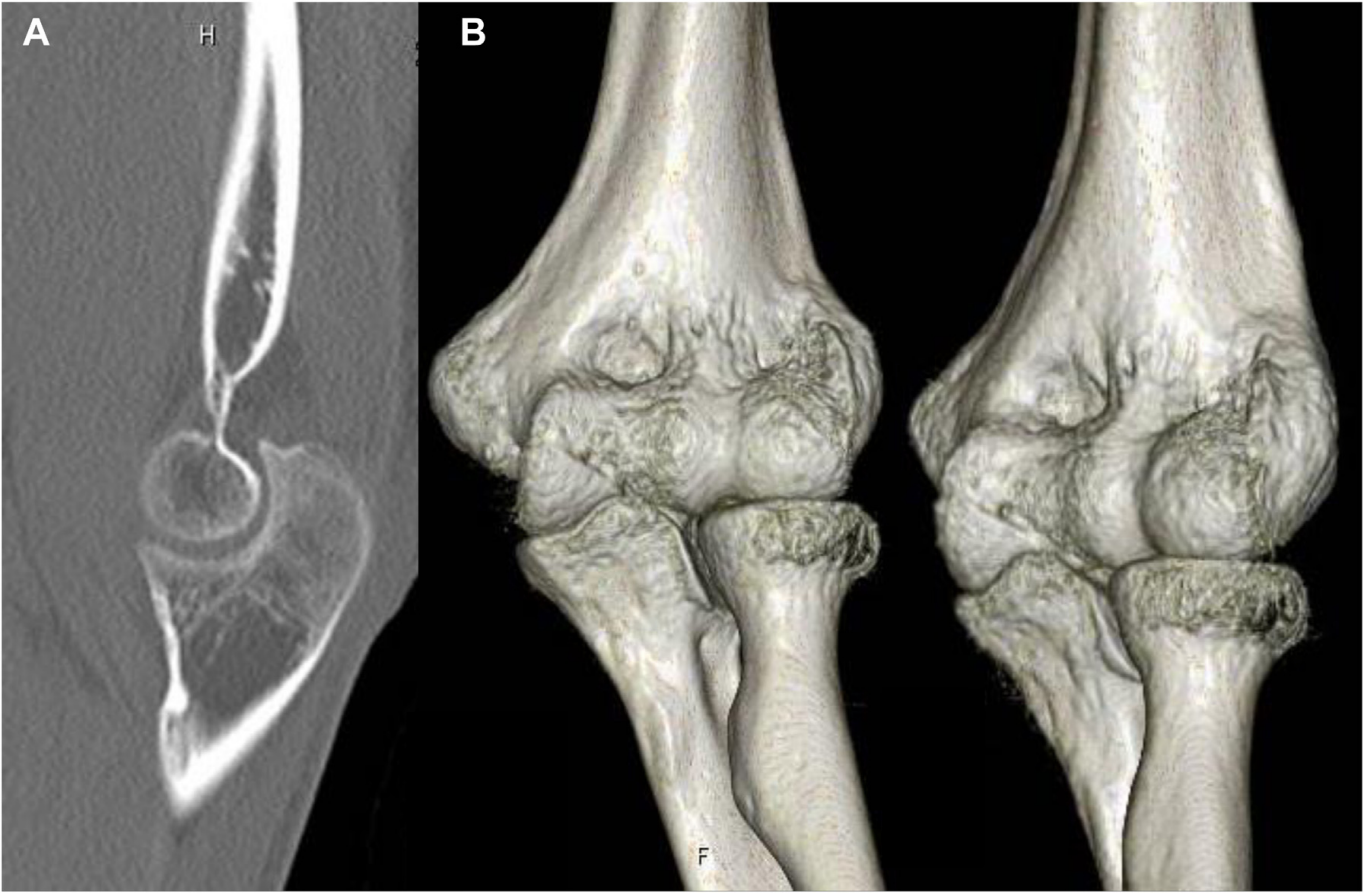
Computed tomography of the elbow joint 6 months after reduction. (**A**) Sagittal image and (**B**) 3-dimensional image. Bony fusion was confirmed with no dislocation.

A 17-year-old male high school student developed left elbow pain after a fall, where he hit his left elbow on the floor while playing volleyball. During a visit to an orthopedic clinic, he was diagnosed with a distal humerus fracture. He was referred to our department the following day. The patient had no past medical history. Physical examination revealed pain and swelling in his left elbow but no paralysis. Due to left elbow pain, the flexion range of motion was restricted to 70°, restricting elbow movement. Plain radiography of the elbow joint showed a coronal shear fracture of the distal humerus, with migration of a bone fragment to the proximal side (Fig. 1). As the fracture involved the capitellum and the trochlea as one piece and comminution in the posterior wall of the distal humerus was absent, we diagnosed Dubberley’s type 2A fracture. The migration distance was measured at 4 mm using computed tomography (Fig. 2). As the fragment was one piece and the posterior wall of the distal humerus was intact, closed reduction was attempted. First, the patient’s left elbow was aspirated to remove the hematoma, and a local anesthetic was injected. Next, we flexed his left elbow to 150°. Additionally, we pronated and supinated his left forearm several times with fluoroscopy. We then assessed the reduction position (Fig. 3) and fixed it with long arm casting at 110° of elbow flexion. After 3 weeks of casting, elbow flexion training was started with splint-restricted extension to 70°. After another week, the patient was allowed full range of motion. At the 6-month follow-up, bony fusion was confirmed with no dislocation noted on computed tomography (Fig. 4). On physical examination, flexion and extension angles of the elbow joint were 135° and −10°, respectively. Pronation and supination angles were similar to that of the unaffected side. The patient showed full recovery as the pain abated.

## Discussion

We report a case of Dubberley’s type 2A coronal shear fracture of the distal humerus treated conservatively, achieving bony fusion with no dislocation and good elbow function. In an intra-articular fracture, operative treatment is generally recommended. However, in cases with nonoperative reduction, conservative treatment should be attempted.

Coronal fractures of the distal humerus involve the capitellum, trochlea, or a combination of both.^4^ Direct axial compression transmitted to the capitellum by the radial head, with the elbow in a semi-flexed position, may create a shear capitellum and trochlea fracture.^9^ Robinson et al^8^ reported 5.7 cases of coronal fractures per 100,000 persons per year with an almost equal male to female ratio. Watts et al^11^ also reported that the annual incidence of distal humeral articular fracture was 1.5 cases per 100,000, with a marked predominance in females. Both studies showed that elderly females were injured due to falls from standing height; however, high-energy falls, such as motor vehicle accidents or injuries while playing sports, were common in males.^8^^,^^11^

Dubberley’s type 2A fractures are the same classification as AO Foundation-Orthopaedic Trauma Association type 13B3.3. Robinson et al reported that most distal humeral metaphyseal fractures were extra-articular (Arbeitsgemeinschaft für Osteosynthesefragen/Orthopaedic Trauma Association (AO/OTA) type A) or complete articular fractures (AO/OTA type C). Only 2 cases of AO/OTA type B3.3 fractures were observed out of 320 fractures of the distal humeral metaphysis.^8^ Guitton et al^2^ reported only 1 case of Dubberley’s type 2A fracture out of 27 fractures of the capitellum and trochlea. In both reports, Dubberley’s type 2A fractures are uncommon.

Studies recommend open reduction and internal fixation for distal humeral fractures because they are almost intra-articular, and anatomical reduction is necessary for elbow motion and stability.^10^ Sequelae of nonanatomic reduction or failed fixation are significant and include articular incongruity, post-traumatic arthrosis, stiffness, and pain.^9^ As regards operative treatment, good outcomes were reported by several authors. However, several complications were confirmed, for example, infection, implant loosening, nerve palsy, and restricted range of motion.^1^^,^^2^^,^^8^ In complex cases like posterior comminution, bone fragments, open fractures, or complications were observed, and elbow function scores became lower than those with isolated capitellum or trochlea fractures.^1^^,^^2^ Robinson et al conducted an observational cohort study with 320 patients and compared bone union rates in operative and nonoperative treatments. There were no significant differences in the nonunion rate.^8^ Furthermore, general anesthesia was administered several times during the preoperative examination.

To the best of our knowledge, this is the first case report of conservative treatment for Dubberley’s type 2A fracture. Nonoperative management is reserved for patients with completely nondisplaced fractures, patients who cannot tolerate anesthesia, or in patients with advanced dementia. In these patients, conservative treatment with early mobilization is appropriate.^5^ There are no reports regarding the conservative treatment of Dubberley’s type 2A fractures because conservative treatment was not recommended; thus, only reports of conservative treatment for Dubberley’s type 1 fractures were found. Open reduction and internal fixation is typically recommended for Dubberley’s type 1 fractures. Puloski et al conservatively treated 7 patients with an isolated fracture of the humeral capitellum. Treatments consisted of closed reduction and arm splint at 90° flexion. Mobilization was started 14 days after the reduction. There were no complications, such as nonunion and conversion to open reduction internal fixation.^7^ Additionally, Ogawa and Shirasawa treated Dubberley’s type 1A fractures by closed reduction after aspiration of joint hematoma with intra-articular anesthesia and casting above the elbow for 18 days. They listed 3 criteria for the conservative treatment of humeral capitellum fractures: (1) the fracture fragment contains intact cortex bone on the posterior aspect of the distal humerus; (2) the fracture fragment is not shattered into many pieces; and (3) after closed reduction, the fracture is confirmed by fluoroscopy to be reduced at its anatomical position with stability.^6^ In the present case, the posterior humeral cortex was intact, with only one bone fragment, and the fracture line spread from the capitellum to the trochlea. After closed reduction, the anatomical position was confirmed by plain radiography. Therefore, the conservative treatment appeared feasible. After anatomical reduction, we fixed a cast above the elbow at 110° of flexion to maintain the reduction position because the bone fragment was stabilized anatomically between the capitellum and posterior humeral cortex in the elbow flexion position. In 90° of flexion, the capitellum would push up the bone fragment proximally, and anatomical reduction could not be maintained. We maintained the cast for 3 weeks and started mobilization with an extension limited splint because elbow extension failed to maintain the reduction position as it pushed the bone fragment to the proximal side of the capitellum. This casting resulted in a stable bone union. No dislocation was seen after 8 weeks of injury.

The limitation of this report is that only a single case is reported. A large sample size is needed to research the outcomes and complications comparing operative and conservative treatments. We could not find any reports regarding conservative treatment for Dubberley’s type 2A fractures in the literature. As Dubberley’s type 2A fractures are rare, case reports may not be published.

In conclusion, the fracture was classified as a Dubberley’s type 2A fracture as there were no comminutions of the posterior distal humerus. Also, only one piece of bone fragment was seen. Additionally, confirmation of anatomical reduction after closed reduction via fluoroscopy was also required. Closed reduction and casting are easy to prepare and can be performed quickly in outpatient care, making them a great option for the treatment of this fracture.

## Conclusion

We present a case where a coronal shear fracture of the distal humerus was treated conservatively and showed good bony union and elbow function. We conclude that conservative treatment is a feasible option for Dubberley’s type 2A fractures.
